# Plasma and intrapulmonary pharmacokinetics of funobactam, a novel diazabicyclooctane β-lactamase inhibitor, administered in combination with imipenem/cilastatin in healthy subjects

**DOI:** 10.1128/aac.00524-26

**Published:** 2026-06-15

**Authors:** Keith Rodvold, Mark Gotfried, Aramayis Kocharyan, Grigor Mamikonyan, Qifeng Shi, Hui Zhao, Ting Hu, Allan Xu, Meijie Le, Jianguo Li

**Affiliations:** 1 University of Illinois Chicago 14681, Chicago, Illinois, USA; 2 Pulmonary Associates, PA 92437, Phoenix, Arizona, USA; 3 Clinartis LLC, Hollywood, Florida, USA; 4 Evopoint Biosciences Co., Ltd. 662696 , Suzhou, China; 5 Keystone Boanalytical, LLC, North Wales, Pennsylvania, USA; 6 Evopoint Biosciences USA, Inc., Massachusetts, USA; Providence Portland Medical Center, Portland, Oregon, USA

**Keywords:** funobactam, XNW4107, intrapulmonary pharmacokinetics, epithelial lining fluid, alveolar macrophages

## Abstract

Funobactam (formerly XNW4107) is a novel non-β-lactam diazabicyclooctane β-lactamase inhibitor with potent direct activity against Ambler classes A, C, and D β-lactamases. Imipenem in combination with funobactam exhibits robust activity against carbapenem-resistant *Enterobacterales*, *Acinetobacter baumannii*, and *Pseudomonas aeruginosa*. This study evaluated the intrapulmonary pharmacokinetics of funobactam, imipenem, and cilastatin in healthy subjects. Twenty participants received a 60 min intravenous infusion of imipenem 500 mg/cilastatin 500 mg combined with funobactam 250 mg every 6 h for five doses. Bronchoalveolar lavage (BAL) was performed at 1, 2, 4, or 6 h after the fifth dose (*n* = 5 per sampling time point) to determine epithelial lining fluid (ELF) and alveolar macrophage (AM) concentrations. The means from each plasma sampling time corresponding to the BAL sampling time were used to estimate the area under the curve from 0 to 6 h (AUC_0–6h_) for plasma, and mean concentrations from BAL sampling times from each cohort were used to estimate AUC_0–6h_ for ELF and AM concentrations. Intrapulmonary penetration was assessed using AUC_0–6h_ ratios of ELF and AM to unbound plasma. The AUC_0–6h_ ratios of ELF to unbound plasma for funobactam, imipenem, and cilastatin were 35.2%, 31.8%, and 23.0%, respectively, and the AUC_0–6h_ ratios of AM to unbound plasma were 36.6%, 16.8%, and 17.9%, respectively. Funobactam penetrated ELF and AM and exhibited higher intrapulmonary penetration than imipenem and cilastatin, supporting the clinical evaluation of funobactam in combination with imipenem for the treatment of nosocomial pneumonia.

This study is registered with ClinicalTrials.gov as NCT04802863.

## INTRODUCTION

Carbapenem-resistant gram-negative bacteria have emerged as a major global public health threat, particularly among hospitalized and critically ill patients. Pathogens such as carbapenem-resistant *Enterobacterales* (CRE), carbapenem-resistant *Acinetobacter baumannii* (CRAB), and carbapenem-resistant *Pseudomonas aeruginosa* (CRPA) are frequently associated with various serious infections, including hospital-acquired bacterial pneumonia (HABP) and ventilator-associated bacterial pneumonia (VABP), with high morbidity, mortality, and limited treatment options (1–4). The primary mechanism driving carbapenem resistance in these organisms is the production of β-lactamases, including Ambler class A carbapenemases (e.g., *Klebsiella pneumoniae* carbapenemase), class C cephalosporinases (AmpC), and class D oxacillinases (OXA-type enzymes), which hydrolyze β-lactam antibiotics and render them ineffective (5–8).

The development of β-lactamase inhibitors (BLIs) has significantly advanced the treatment of infections caused by resistant gram-negative bacteria by restoring the activity of partner β-lactam agents. Diazabicyclooctane (DBO) BLIs, such as avibactam and relebactam, have demonstrated broad inhibitory activity against class A and C enzymes and some class D enzymes, leading to the successful clinical introduction of combination therapies such as ceftazidime-avibactam and imipenem-relebactam (9–11). These combinations have improved clinical outcomes in patients with infections caused by carbapenem-resistant pathogens and represent important therapeutic advances. Nevertheless, the continued emergence of resistance and the limited activity of existing BLIs against certain β-lactamases highlight the need for novel inhibitors with improved potency and broader spectrum (12, 13).

Funobactam (formerly XNW4107) is a novel, non-β-lactam DBO β-lactamase inhibitor with potent inhibitory activity against Ambler class A, C, and D β-lactamases (14–16). When combined with imipenem, funobactam has demonstrated robust *in vitro* activity against CRE, CRAB, and CRPA, including isolates resistant to currently available β-lactam/β-lactamase inhibitor combinations. In particular, imipenem in combination with funobactam exerts strong antibiotic activity against *A. baumannii*. The addition of funobactam to imipenem *in vitro* restores the activity of imipenem, such that the MIC_90_ from a collection of globally diverse contemporary *A. baumannii* clinical isolates (*n* = 500) decreases from 128 mg/L to 2 mg/L in the presence of funobactam (held constant at 8 mg/L) (16). The combination of imipenem/cilastatin with funobactam (IMI-FUN) is being developed to treat infections including those caused by CRE, CRAB, and CRPA. The U.S. Food and Drug Administration (FDA) has granted the qualified infectious disease product designation and fast track status to IMI-FUN for the treatment of infections including HABP/VABP and complicated urinary tract infection.

The objectives of this study were to (i) determine and compare plasma, epithelial lining fluid (ELF), and alveolar macrophage (AM) concentrations of funobactam, imipenem, and cilastatin after five repeated intravenous dose administrations of imipenem 500 mg/cilastatin 500 mg and funobactam 250 mg every 6 h, (ii) characterize the plasma pharmacokinetics of funobactam, imipenem, and cilastatin, and (iii) assess the safety and tolerability of concurrent intravenous administration of imipenem 500 mg/cilastatin 500 mg and funobactam 250 mg every 6 h for five consecutive doses in healthy adult subjects (ClinicalTrials registration no. NCT04802863). This work was accepted as a poster presentation at the European Society of Clinical Microbiology and Infectious Diseases conference, Munich, Germany, April 2026.

## RESULTS

Twenty-one adult subjects (14 males, 7 females; age: 19–45 years) were enrolled in the study and included in the safety population. One subject discontinued the study drug due to a serious adverse event (SAE). Twenty subjects completed the required study procedures and were included in the pharmacokinetic population (Table S1).

**Fig 1:**
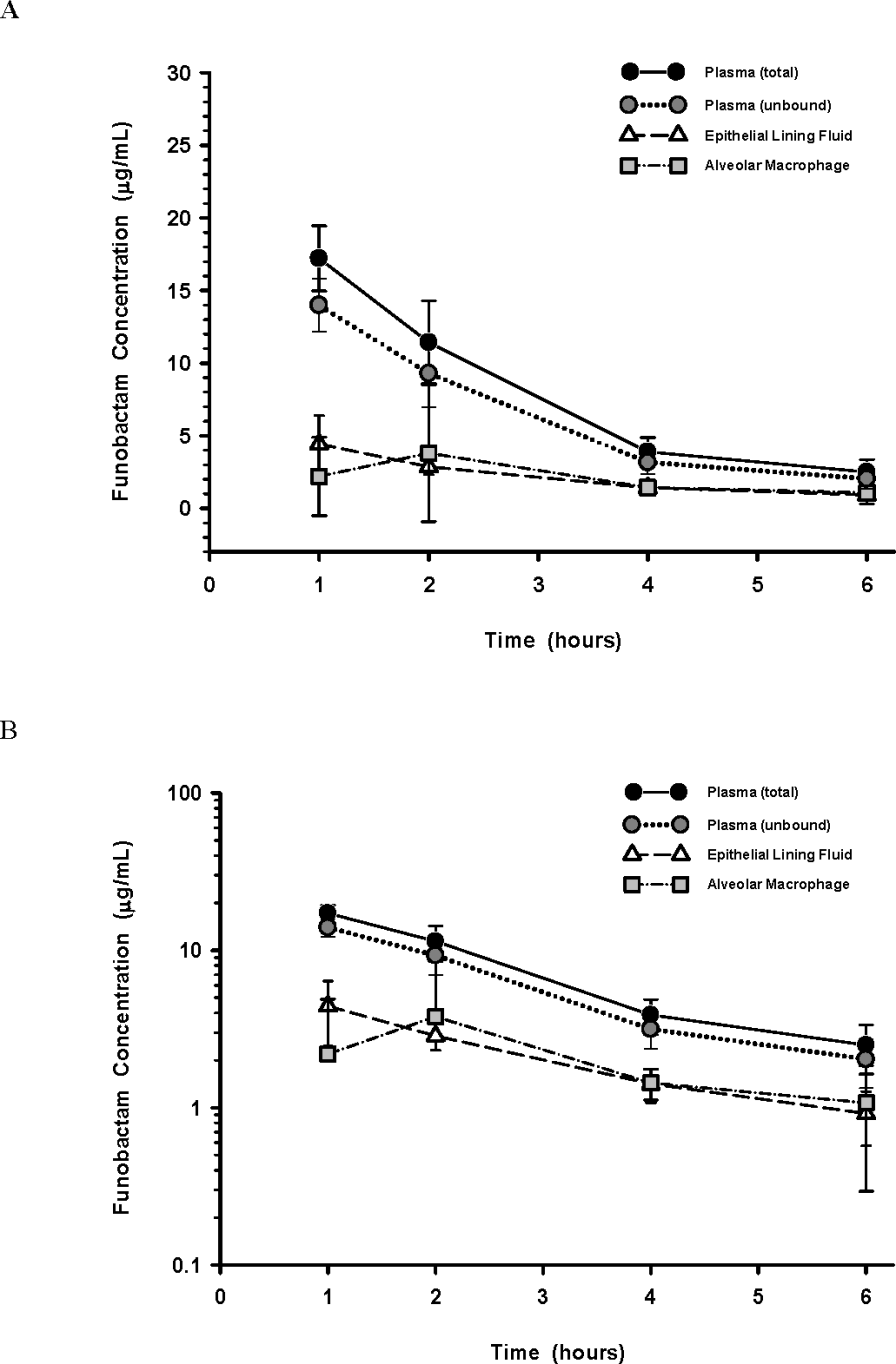
Mean (± standard deviation [SD]) concentration versus time profiles of funobactam in total plasma, unbound plasma, ELF, and AM at the scheduled BAL sampling times after the fifth intravenous infusion of imipenem 500 mg/cilastatin 500 mg in combination with funobactam 250 mg every 6 h. The *y*-axis of panel **A** is a linear scale, and that of panel **B** is a semi-log scale.

**Fig 2:**
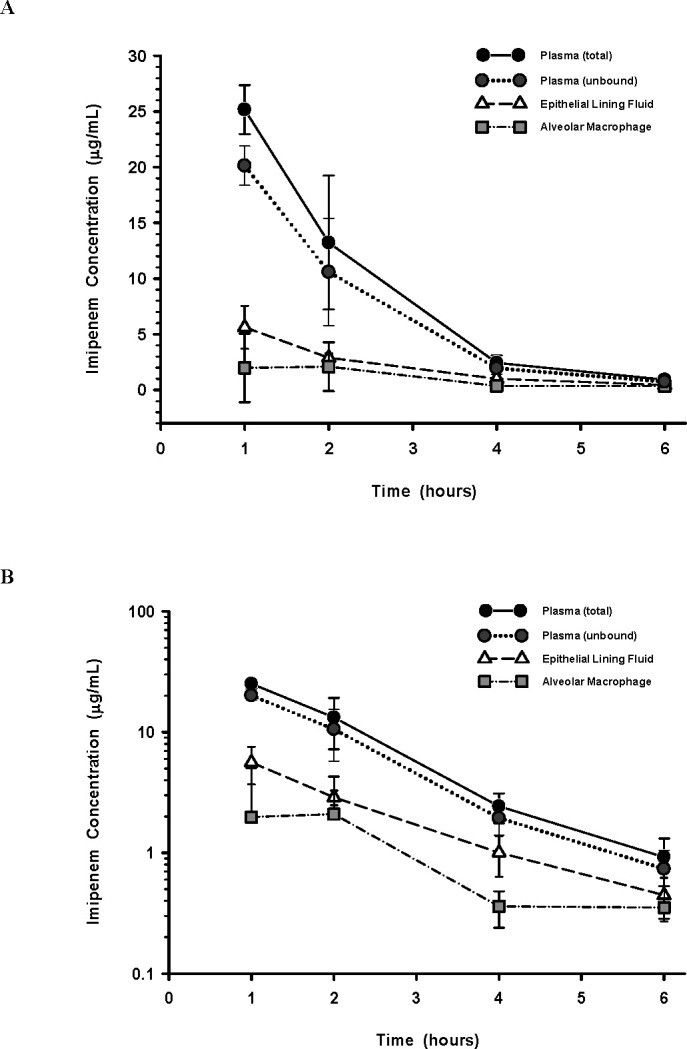
Mean (± SD) concentration versus time profiles for imipenem in total plasma, unbound plasma, ELF, and AM at the scheduled BAL sampling times after the fifth intravenous infusion of imipenem 500 mg/cilastatin 500 mg in combination with funobactam 250 mg every 6 h. The *y*-axis of panel **A** is a linear scale, and that of panel **B** is a semi-log scale.

**Fig 3:**
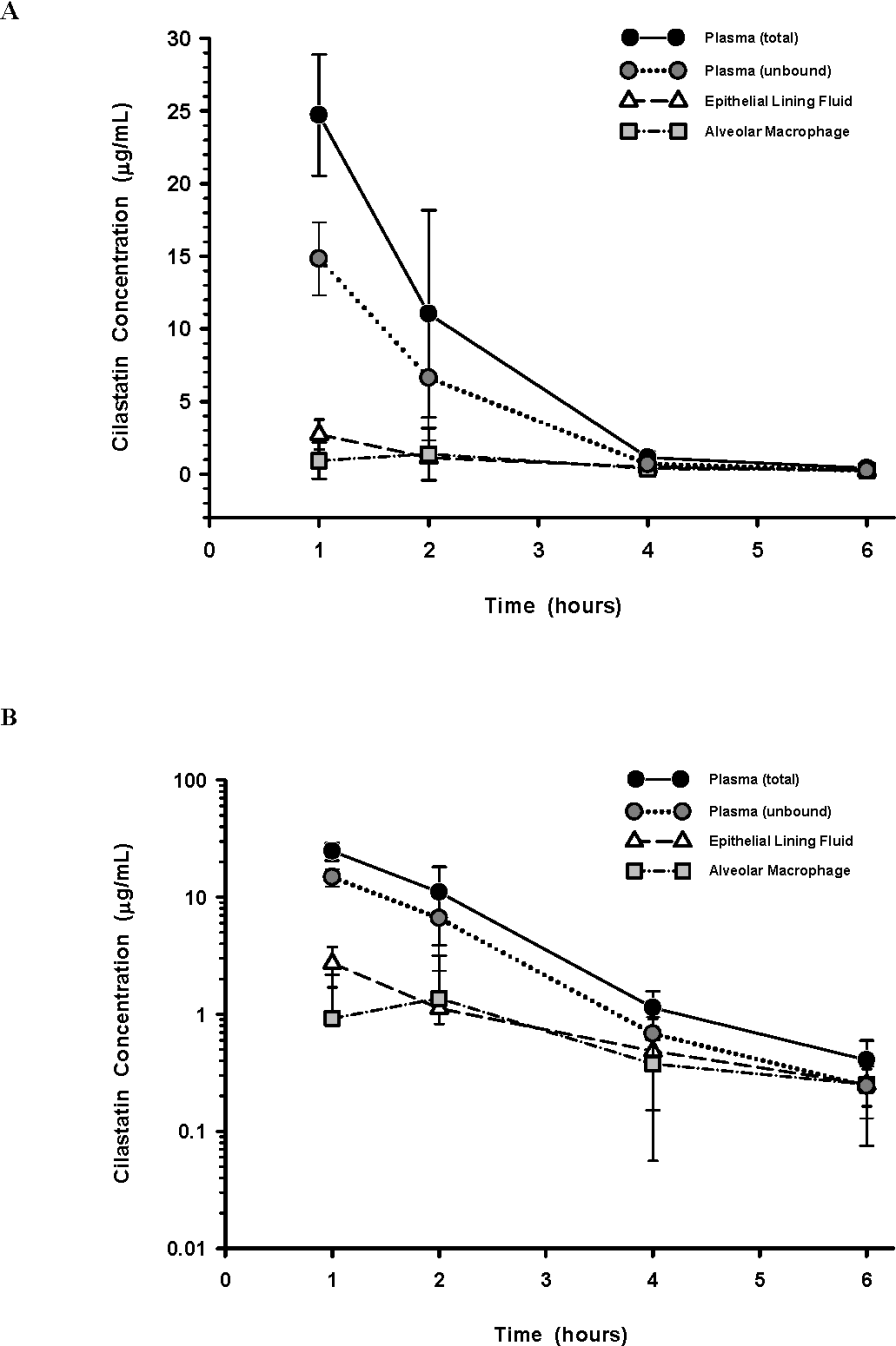
Mean (± SD) concentration versus time profiles for cilastatin in total plasma, unbound plasma, ELF, and AM at the scheduled BAL sampling times after the fifth intravenous infusion of imipenem 500 mg/cilastatin 500 mg in combination with funobactam 250 mg every 6 h. The *y*-axis of panel **A** is a linear scale, and that of panel **B** is a semi-log scale.

Following five consecutive intravenous dose administrations of imipenem 500 mg/cilastatin 500 mg in combination with funobactam 250 mg every 6 h, the concentration-time courses in total plasma, unbound plasma, ELF, and AM are shown in Fig. 1 for funobactam, Fig. 2 for imipenem, and Fig. 3 for cilastatin. Concentrations of funobactam, imipenem, and cilastatin in plasma and ELF exhibited a similar time course and pattern. The mean time (*T*_max_) to maximum concentrations (*C*_max_) in plasma and ELF occurred at the 1 h sampling time (Tables S2 to S4) and suggests limited hysteresis between plasma and extracellular concentrations. In contrast, the mean AM concentration-time courses of funobactam, imipenem, and cilastatin had *T*_max_ values of 2 h, later than that of 1 h for the mean plasma concentration-time courses.

The plasma pharmacokinetic parameters of funobactam, imipenem, and cilastatin are shown in Table 1, with the mean half-life (t_1/2_) of 2.74 h for funobactam, longer than that for imipenem and cilastatin (1.39 and 1.17 h, respectively). The means and SDs of the plasma concentrations obtained before the third, fourth, and fifth doses of funobactam (2.06 ± 0.74 µg/mL, 2.46 ± 0.73 µg/mL, and 2.57 ± 0.72 µg/mL, respectively), imipenem (0.85 ± 0.31 µg/mL, 0.90 ± 0.34 µg/mL, and 0.90 ± 0.32 µg/mL, respectively), and cilastatin (0.44 ± 0.22 µg/mL, 0.43 ± 0.21 µg/mL, and 0.42 ± 0.22 µg/mL, respectively) were similar in value and confirmed that steady-state conditions were established by the fifth dose.

**TABLE 1:** Pharmacokinetic parameters of funobactam, imipenem, and cilastatin in plasma after the fifth intravenous infusion of imipenem 500/cilastatin 500 mg in combination with funobactam 250 mg every 6 h^*a*^^,^^*c*^^,^^*d*^

	*C*_max_ (µg/mL)	*T*_max_ (h)	*C*_min_ (µg/mL)^*b*^	AUC_0–6h_ (µg•h/mL)	AUC_0–∞_ (µg•h/mL)	CL (L/h)	V_z_ (L)	t_1/2_ (h)
Funobactam	16.64 ± 1.87	1.02 (1.00, 2.00)	2.45 ± 0.71	41.70 ± 6.63	52.27 ± 10.47	6.16 ± 1.09	23.96 ± 3.71	2.74 ± 0.43
Imipenem	25.15 ± 2.93	1.02 (1.00, 2.00)	0.93 ± 0.35	42.87 ± 6.45	44.82 ± 7.10	11.95 ± 2.00	23.78 ± 3.89	1.39 ± 0.18
Cilastatin	22.80 ± 3.41	1.02 (1.00, 2.00)	0.47 ± 0.25	33.37 ± 6.81	34.23 ± 7.22	15.63 ± 3.46	25.75 ± 5.19	1.17 ± 0.22

^*a*^ 20 parameter values for each listing.

^*b*^ The *C*_min_ is defined as the lowest plasma concentration during the 6 h dosing interval and after the fifth dose was administered.

^*c*^ Arithmetic mean (± SD) is presented for *C*_max_, *C*_min_, AUC_0–6h_, AUC_0–∞_, CL, V_Z_, and t_1/2_.

^*d*^ Median (minimum, maximum) is reported for *T*_max_.

The penetration into the extracellular space, based on the mean ratios of ELF to the corresponding unbound plasma concentrations across the four sampling time points (1, 2, 4, and 6 h), ranged from 0.309 to 0.452 for funobactam, 0.279 to 0.613 for imipenem, and 0.183 to 0.812 for cilastatin, respectively (Table 2). The overall penetration into the lung extracellular space, based on the ratio of area under the concentration curve from 0 to 6 h (AUC_0–6h_) of ELF to the AUC_0–6h_ of unbound plasma, was 35.2% for funobactam, 31.82% for imipenem, and 23.0% for cilastatin, respectively (Table 3).

**TABLE 2:** Ratios of ELF and AM to total and unbound plasma concentrations of funobactam, imipenem, and cilastatin^*g*^

	BAL sampling time	ELF to plasma (total)	ELF to plasma (unbound)^*a*^	AM to plasma (total)	AM to plasma (unbound)^*a*^
Funobactam	1 h^*b*^	0.251 ± 0.094	0.309 ± 0.116	0.126 ± 0.147	0.156 ± 0.181
	2 h^*b*^	0.264 ± 0.080	0.325 ± 0.099	0.398 ± 0.503	0.490 ± 0.619
	4 h^*b*^	0.368 ± 0.050	0.452 ± 0.062	0.425 ± 0.145	0.522 ± 0.179
	6 h^*b*^	0.365 ± 0.040	0.449 ± 0.050	0.472 ± 0.289	0.580 ± 0.356
Imipenem	1 h^*b*^	0.223 ± 0.077	0.279 ± 0.097	0.086 ± 0.129	0.108 ± 0.161
	2 h^*b*^	0.241 ± 0.072	0.302 ± 0.090	0.209 ± 0.220	0.261 ± 0.275
	4 h^*b*^	0.417 ± 0.120	0.521 ± 0.150	0.155 ± 0.066^*c*^	0.194 ± 0.082^*c*^
	6 h^*b*^	0.491 ± 0.051	0.613 ± 0.064	0.461 ± 0.064^*d*^	0.577 ± 0.079^*d*^
Cilastatin	1 h^*b*^	0.110 ± 0.043	0.183 ± 0.071	0.043 ± 0.057	0.071 ± 0.095
	2 h^*b*^	0.123 ± 0.048	0.205 ± 0.081	0.179 ± 0.216	0.298 ± 0.360
	4 h^*b*^	0.437 ± 0.312	0.729 ± 0.520	0.323 ± 0.174^*c*^	0.538 ± 0.290^*c*^
	6 h^*b*^	0.487 ± 0.125^*e*^	0.812 ± 0.208^*e*^	0.667 ± 0.336^*f*^	1.111 ± 0.560^*f*^

^*a*^ The value used for the unbound fraction of funobactam, imipenem, and cilastatin in plasma are 0.81, 0.80, and 0.60, respectively.

^*b*^ Five subjects per sampling time.

^*c*^ Based on four of five subjects had cell pellet concentrations above the lower limit of quantification (LLOQ) of 2 ng/mL.

^*d*^ Based on two of five subjects had cell pellet concentrations above the LLOQ of 2 ng/mL.

^*e*^ Based on three of five subjects had BAL concentrations above the LLOQ of 4 ng/mL.

^*f*^ Based on three of five subjects had cell pellet concentrations above the LLOQ of 2 ng/mL.

^*g*^ Arithmetic mean (± SD) is presented.

**TABLE 3:** Ratio of the AUC_0–6h_ of ELF and AM to the AUC_0–6h_ total and unbound plasma of funobactam, imipenem, and cilastatin

Treatment	Ratio of the AUC_0–6h_ of ELF to AUC_0–6h_ of plasma (total)	Ratio of the AUC_0–6h_ of ELF to AUC_0–6h_ of plasma (unbound)^*a*^^,^^*b*^^,^^*c*^	Ratio of the AUC_0–6h_ of AM to AUC_0–6h_ of plasma (total)	Ratio of the AUC_0–6h_ of AM to AUC_0–6h_ of plasma (unbound)^*a*^^,*b*,*c*^
Funobactam	0.286	0.352	0.297	0.366
Imipenem	0.254	0.318	0.134	0.168
Cilastatin	0.138	0.230	0.107	0.179

^*a*^ The value used for the unbound fraction of funobactam in plasma was 0.813.

^*b*^ The value used for the unbound fraction of imipenem in plasma was 0.80.

^*c*^ The value used for the unbound fraction of cilastatin in plasma was 0.60.

The penetration into the intracellular space based on mean ratios of AM to the corresponding unbound plasma concentrations across the four sampling time points ranged from 0.156 to 0.58 for funobactam, 0.108 to 0.577 for imipenem, and 0.071 to 1.11 for cilastatin, respectively (Table 2). The overall penetration into the lung intracellular space based on the AUC_0–6h_ ratio of AM to unbound plasma was 36.6% for funobactam, 16.82% for imipenem, and 17.9% for cilastatin, respectively (Table 3).

Of 21 subjects enrolled in the study, 4 subjects (19.0%) experienced a total of seven treatment-emergent adverse events (TEAEs) over the course of the study. Of the seven reported TEAEs, four events (sinus congestion, nausea, flushing, pericarditis) were considered possibly related to the study drug. Three events (rash, headache, arthropod bite) were considered unrelated or unlikely to be related to study drug administration. Five TEAEs were mild in severity, one TEAE was moderate, and one TEAE was severe. The one adverse event (pericarditis) of severe intensity was considered an SAE leading to the subject’s discontinuation of study treatment. This SAE was considered possibly related to the study drug and resolved at the follow-up visit. No clinically significant changes were observed in hematology, biochemistry, coagulation, and urinalysis parameters, in vital signs and electrocardiogram (ECG) parameters, or physical examination findings during the study.

## DISCUSSION

This study characterized the plasma and intrapulmonary pharmacokinetics of funobactam, a novel diazabicyclooctane β-lactamase inhibitor, administered in combination with imipenem/cilastatin in healthy adult subjects. Following repeated intravenous dosing, funobactam demonstrated measurable and sustained penetration into both ELF and AM, with ELF and AM exposure (AUC_0–6h_) corresponding to 35.2% and 36.6% of unbound plasma, respectively. Importantly, the ELF concentration-time profiles of funobactam paralleled plasma exposure and achieved peak concentrations at similar time points in ELF to those in plasma, indicating rapid equilibration between plasma and the pulmonary epithelial lining fluid. These findings demonstrate that funobactam achieves clinically meaningful intrapulmonary exposure at relevant sites of infection and support its development in combination with imipenem/cilastatin for the treatment of bacterial pneumonia, including infections caused by carbapenem-resistant gram-negative pathogens.

This study demonstrated a mean t_1/2_ of 2.74, 1.39, and 1.17 h for funobactam, imipenem, and cilastatin, respectively, which is consistent with previously reported pharmacokinetic values in the first human study in healthy volunteers (17).

Adequate β-lactam and β-lactamase inhibitor penetration into the lung is essential for effective treatment of nosocomial infections such as HABP/VABP, as the ELF represents the primary extracellular site of infection for most bacterial respiratory pathogens (18–20). The observed ELF penetration ratio of funobactam relative to unbound plasma (35.2%) is consistent with those reported for other DBO inhibitors. In an intrapulmonary pharmacokinetic study, relebactam administered with imipenem/cilastatin demonstrated ELF penetration of approximately 54% relative to unbound plasma (21). The differences in the ELF penetration are most likely due to the limited and different ELF sampling times (1, 2, 4, and 6 h in this study in contrast to 0.5, 1, 1.5, and 3 h in the relebactam study). Similarly, avibactam has been shown to achieve ELF penetration ratios of approximately 30 to 50% in healthy subjects (22). These findings collectively indicate that DBO β-lactamase inhibitors distribute effectively into ELF and that funobactam exhibits pulmonary penetration characteristics consistent with this class of inhibitors. Importantly, the ELF exposure relative to unbound plasma exposure of funobactam (35.2%) was comparable to that of imipenem (31.8%), supporting the pharmacokinetic suitability of the combination and ensuring adequate inhibitor concentrations at the site of infection to protect imipenem from enzymatic degradation.

In addition to extracellular ELF exposure, funobactam demonstrated substantial penetration into alveolar macrophages, with an AUC_0–6h_ ratio of AM to unbound plasma of 36.6%, approximately twice as much as that observed for imipenem (16.8%) and cilastatin (17.9%). Although β-lactams and their inhibitors primarily target extracellular pathogens, intracellular penetration may contribute to therapeutic efficacy against facultative intracellular organisms and may enhance overall pulmonary drug distribution (23, 24). The delayed *T*_max_ observed in AM relative to plasma and ELF suggests intracellular uptake and distribution kinetics are consistent with passive diffusion and cellular accumulation mechanisms described for other β-lactam and β-lactamase inhibitor combinations (21, 22). These findings further support the ability of funobactam to achieve pharmacologically relevant concentrations within key pulmonary compartments.

The parallel concentration-time profiles of funobactam in plasma and ELF and the absence of significant hysteresis between compartments indicate rapid and predictable pulmonary distribution. These pharmacokinetic characteristics are consistent with those reported for relebactam and other DBO inhibitors and suggest that plasma pharmacokinetics may serve as a reasonable surrogate for estimating pulmonary exposure (21, 22, 25). Furthermore, the elimination half-life of funobactam (2.74 h) was longer than that of imipenem (1.39 h), supporting sustained inhibitor exposure during the dosing interval and ensuring continued protection of imipenem from hydrolysis by β-lactamases.

From a clinical perspective, achieving sufficient β-lactamase inhibitor concentrations at the site of infection is critical for restoring the antibacterial activity of the partner β-lactam. Funobactam has demonstrated potent *in vitro* inhibitory activity against Ambler class A, C, and D β-lactamases, including those produced by carbapenem-resistant *Enterobacterales, A. baumannii, and P. aeruginosa* (14–16). The intrapulmonary exposure observed in this study supports the ability of funobactam to penetrate in the lung and thereby have the potential to restore imipenem activity against resistant pathogens. These findings are particularly important given the high morbidity and mortality associated with HABP and VABP caused by carbapenem-resistant organisms and the limited availability of effective treatment options (1–4, 26).

The safety profile observed in this study was generally favorable and consistent with previous clinical experience with β-lactam and β-lactamase inhibitor combinations. Most adverse events were mild or moderate in severity, and no clinically meaningful changes were observed in laboratory parameters, vital signs, or ECG findings. One serious adverse event of pericarditis was reported and resolved without sequelae. Overall, these findings suggest that repeated dosing of funobactam in combination with imipenem/cilastatin was generally well tolerated in healthy subjects.

This study has several strengths, including the use of serial bronchoalveolar lavage to directly measure ELF and AM concentrations and the evaluation of pharmacokinetics following repeated dosing to steady-state conditions. These design elements provide a robust assessment of pulmonary drug exposure and enhance the clinical relevance of the findings. However, several limitations should be acknowledged. First, the study was conducted in healthy subjects, and intrapulmonary pharmacokinetics may differ in critically ill patients due to altered physiology, inflammation, or changes in drug distribution. Second, the sample size at each BAL time point was limited, which may contribute to variability in penetration estimates. Finally, clinical efficacy was not evaluated in this study, and further clinical trials in patients with pneumonia are necessary to confirm the therapeutic relevance of these pharmacokinetic findings.

In conclusion, funobactam demonstrated favorable plasma and intrapulmonary pharmacokinetic properties when administered in combination with imipenem/cilastatin. Funobactam achieved clinically relevant concentrations in epithelial lining fluid and alveolar macrophages, with pulmonary penetration comparable to or exceeding that of imipenem. These findings support the pharmacokinetic suitability of funobactam in combination with imipenem/cilastatin for the treatment of pulmonary infections and provide further evidence supporting the clinical development of imipenem/cilastatin-funobactam as a promising therapeutic option for the treatment of serious respiratory infections caused by carbapenem-resistant gram-negative pathogens.

## MATERIALS AND METHODS

### Study design

This was a phase 1, open-label, single-center safety and PK study in healthy adult subjects between 18 and 55 years of age (both inclusive).

Twenty subjects were planned to receive a total of five doses of funobactam 250 mg in combination with imipenem 500 mg/cilastatin 500 mg via intravenous infusion administered every 6 h, with each administration infused over 60 min. Discontinued subjects could have been replaced on a case-by-case basis per sponsor’s discretion. Subjects who met inclusion and exclusion criteria were confined to the clinical site from day −1 (1 day prior to start of dosing) and remained in the clinic through completion of all scheduled procedures, until after collection of the last pharmacokinetic sample, and completion of bronchoscopy procedures along with safety evaluations.

### Study population

Subjects must meet the following inclusion criteria to be eligible for participation in this study: (i) adult males or female subjects, between 18 and 55 years of age (both inclusive) at the time of screening; (ii) BMI ≥18.5 and ≤32 (kg/m^2^) and weight between 55.0 and 100.0 kg (both inclusive); (iii) medically healthy without clinically significant abnormalities as assessed by the investigator based on screening medical history, physical examination, vital signs, 12-lead ECG, hematology, biochemistry, coagulation, and urinalysis; (iv) forced expiratory volume in 1 s of at least 80% of predicted value at screening; (v) willing and able to provide written informed consent; (vi) willing and able to comply with all study assessments and adhere to the protocol schedule; (vii) have suitable venous access for blood sampling; (viii) non-smoker (with no use of other tobacco, nicotine, or marijuana-containing products, in any form), as documented by history (no nicotine or marijuana use within 3 months prior to screening); (ix) negative urine drug, alcohol, or cotinine testing at screening and check-in (day −1); (x) if female, must be of non-childbearing potential based on at least one of the following criteria: (a) post-menopausal status defined as amenorrhea for at least 12 months prior to the first dosing in the absence of any exogenous hormonal treatments and follicle-stimulating hormone levels in the laboratory-defined post-menopausal range; (b) subject report of surgical sterilization (i.e., bilateral tubal ligation, hysterectomy, bilateral oophorectomy/salpingectomy); or, if female of childbearing potential, willingness to abstain from sexual activity that could lead to pregnancy (i.e., heterosexual vaginal intercourse), or *in vitro* fertilization, from the time of screening throughout the course of the study, or agreement to use a highly effective method of contraception throughout the study and for 30 days after the last study drug administration, including one or more of the following methods: (c) an approved hormonal contraceptive, including oral, implantable, transdermal, or injectable intravaginal contraceptive used consistently for at least 1 month prior to first dosing; (d) an intrauterine device or Essure procedure; (e) history of bilateral tubal ligation/cauterization; (f) dual barrier contraception including any of the following in combination: (i) female condom, contraceptive sponge, diaphragm, or cervical cap with spermicide (cream, spray, gel, suppository, sponge, or polymer film); (ii) male sexual partner who agrees to use a male condom with spermicide in addition to the female subject’s use of spermicide (cream, spray, gel, suppository, sponge, or polymer film); (iii) male sexual partner who has been vasectomized for at least 3 months prior to the screening visit and who has obtained a follow-up negative sperm count; (xi) if male, agree to be sexually abstinent or agree to use two approved methods of contraception when engaging in sexual activity from screening until 90 days following the last administration of the study drug, and to not donate sperm during this same period of time. In the event that the sexual partner is surgically sterile, contraception is not necessary; (xii) ability and willingness to abstain from alcohol, caffeine, xanthine-containing beverages or food (coffee, tea, chocolate, and caffeine-containing sodas, colas, etc.), or products containing any of these from 72 h prior to study drug administration until discharge from the clinical unit.

Subjects must be excluded from participation in the study if there was evidence of any of the following at screening or confinement, as appropriate: (i) history or presence of significant oncologic, cardiovascular, pulmonary, hepatic, renal, hematological, gastrointestinal, endocrine, immunologic, dermatologic, vascular, or neurological disease, including any acute illness or surgery within the past 3 months determined by the investigator to be clinically relevant; (ii) recent history (within 6 months) of known or suspected *Clostridium difficile* infection; (iii) history of seizure disorder; (iv) positive testing for human immunodeficiency virus antibody, hepatitis B surface antigen, or hepatitis C antibody; (v) positive RT-PCR testing for severe acute respiratory syndrome coronavirus 2 (SARS-CoV-2) at screening; (vi) presence of the following symptoms at screening or within 14 days prior to screening or check-in (day −1): (a) fever, chills, or sweats (temperature of 38°C/100.4°F or higher); (b) difficulty breathing; (c) cough; (d) sore throat; (e) new or recent loss of taste or smell; (f) nausea, vomiting, or diarrhea; (vii) close contact with anyone who tested positive for SARS-CoV-2 infection within 14 days prior to screening or check-in (day −1); (viii) ECG with QTcF interval duration equal to or greater than 450 ms for males and 470 ms for females obtained after at least 5 min in a supine or semi-supine position at quiet rest at screening or check-in (day −1); (ix) subjects who have any of the following abnormalities in laboratory values at screening or prior confinement, including (a) white blood cell count <3,000/mm^3^, hemoglobin <11 g/dL; (b) absolute neutrophil count <1,200/mm^3^, platelet count <120,000/mm^3^; (c) alanine aminotransferase and aspartate aminotransferase greater than 1.5× the upper limit of normal for the reference laboratory; (x) history of substance abuse or alcohol abuse defined as an average daily intake of greater than 3 units, or an average weekly intake of greater than 21 units (1 unit is equivalent to one can or bottle [12 oz] of beer, or one measure [1.5 oz] of spirits, or one glass [5 oz] of wine) within the previous 5 years; (xi) use of prescription medications (with the exception of hormone replacement therapy and contraceptives listed in inclusion criterion #10), including nonsteroidal anti-inflammatory drugs, sucralfate, or herbal preparations within 7 days before check-in (day −1), or use of over-the-counter medications, acetaminophen (>2 g/day), vitamins, or supplements (including fish liver oils) within 7 days before check-in (day −1), or probenecid or valproic acid within 30 days before check-in (day −1); (xii) history of hypersensitivity to β-lactam antibiotics or drugs that include sulfobutylether β-cyclodextrin sodium as an excipient (e.g., Tegretol, Vfend, Geodon, and Noxafil); (xiii) history of significant multiple and/or severe allergies (including latex allergy), anaphylactic reaction, or significant prescription drug, non-prescription drug, or food intolerance; (xiv) donation of blood or plasma within 30 days prior to check-in (day −1), or loss of whole blood of more than 500 mL within 30 days prior to check-in (day −1), or receipt of a blood transfusion within 1 year of study enrollment; (xv) participation in another investigational clinical trial within 30 days prior to screening; (xvi) a female who is pregnant or breastfeeding; (xvii) any other condition or prior therapy, which, in the opinion of the investigator, would make the volunteer unsuitable for this study, including inability to cooperate fully with the requirements of the study protocol or likely to be non-compliant with any study requirements.

### Drug administration

Subjects received five doses of funobactam 250 mg in combination with imipenem 500 mg/cilastatin 500 mg via intravenous infusion, administered every 6 h (±10 min), with each administration infused over 60 min. The infusions of funobactam and imipenem/cilastatin were initiated at the same time. The start and end time of each infusion were recorded in the eCRF. A window of ±3 min was utilized for recording deviations in the infusion duration. The sponsor (Evopoint Biosciences USA, Inc., Concord, MA) provided adequate supplies of funobactam and Primaxin for use during the study.

### Blood sample collection

Blood samples for determining plasma funobactam and imipenem/cilastatin concentrations were collected at the following time points: before administration of the first, third, and fourth doses, and prior to (0 h) and at the end of infusion (1 h), and at 1.5, 2, 4, 6, 8, and 12 h after start of the fifth intravenous dose of funobactam 250 mg in combination with imipenem 500 mg/cilastatin 500 mg.

### Bronchoscopy and BAL

Each subject was assigned to one of four bronchoscopy sampling times according to bronchoscopy suite scheduling times and subject availability. The bronchoscopy sampling times were at 1, 2, 4, or 6 (±10 min) h after the fifth dose administration of funobactam 250 mg and imipenem 500 mg/cilastatin 500 mg. Blood samples (approximately 5 mL) for determining urea concentration and blood PK samples were obtained during the bronchoscopy procedure. The collection process of the blood urea sample and blood PK sample started just prior to collection of the second aspirate of BAL.

### Determination of plasma concentrations of funobactam, imipenem, and cilastatin

The concentrations of funobactam, imipenem, and cilastatin in plasma were determined by a validated liquid chromatography with tandem mass spectrometry (LC-MS/MS) assay operated in the positive ion mode (method number M201105) performed at Keystone Bioanalytical, Inc. (North Wales, PA). A total of 224 plasma samples were assayed for funobactam, imipenem, and cilastatin concentrations. A total of 23 samples were selected for incurred sample assay reproducibility (ISR) testing, and all of them met acceptance criteria, with at least two-thirds of the repeat results and original results falling within 20% of the mean of the two values. For all analytical runs, fortified calibration standards and quality controls (QCs) met acceptance criteria of within ±15% of the nominal concentration (±20% at the LLOQ).

In brief, the method used protein precipitation (1:1 acetonitrile/methanol is used as the solvent) to isolate funobactam, imipenem, cilastatin, and the internal standards (funobactam-15N-13C3 [Bellen, lot no. 60401-My-026], imipenem-d4 [ALSA CHIM, lot no. MS-ALS-17-241-P2], and cilastatin-15N-d3 [Cayman, lot no. 0573551]). Pre-treated human K_2_EDTA plasma was used as a blank matrix. Human K_2_EDTA plasma is pretreated by mixing one part of stabilizer (25:25:50 ethylene glycol/water/1 M pH 6.0 MES) with one part of human K_2_EDTA plasma (1:1, vol/vol). Pre-treated human K_2_EDTA plasma was spiked with the appropriate concentration of working standard solution to give the calibration standards and QC samples. After shaking and centrifuging, 10 μL of the supernatant was transferred into an injection vial containing 1.5 mL of water, and then the vial was capped and vortexed for 30 s. A total of 25 μL–50 μL injection volume was used for LC-MS/MS analysis.

The standard curves were linear for funobactam (*R*^2^ ≥ 0.9966) over the concentration ranges of 25 to 12,500 ng/mL. The standard curves were linear for imipenem (*R*^2^ ≥ 0.9948) and cilastatin (*R*^2^ ≥ 0.9949) over the concentration ranges of 50 to 25,000 ng/mL. The respective precision and accuracy for funobactam QC samples were 4.36% and 7.24% at 75 ng/mL, 5.76% and 4.84% at 3,750 ng/mL, and 4.15% and 0.39% at 8,750 ng/mL. The respective precision and accuracy for imipenem QC samples were 2.76% and 2.22% at 150 ng/mL, 5.63% and 2.80% at 7,500 ng/mL, and 2.80% and −2.00% at 17,500 ng/mL. The respective precision and accuracy for cilastatin QC samples were 4.38% and 5.03% at 150 ng/mL, 4.39% and 4.24% at 7,500 ng/mL, and 2.13% and −0.51% at 17,500 ng/mL. The LLOQ for funobactam was 25 ng/mL and the LLOQ for imipenem and cilastatin was 50 ng/mL. Reported concentrations were multiplied by a factor of 2 to account for the 1:1 dilution of plasma samples with stabilizer.

### Determination of BAL fluid and cell pellet concentrations of funobactam, imipenem, and cilastatin

The determinations of funobactam, imipenem, and cilastatin in BAL fluid supernatants and reconstituted cell pellet suspensions were assayed with LC-MS/MS using a Turbo ion spray interface operated in positive ion mode at Keystone Bioanalytical (North Wales, PA) (report number 210511). A total of 20 BAL fluid and 20 cell pellet samples were assayed for funobactam, imipenem, and cilastatin concentrations. A total of five samples for BAL fluid (25.0%) were selected for ISR testing, and the calculated assay variability values were within 20%.

In brief, the method used protein precipitation (1:1 acetonitrile/methanol is used as solvent) to isolate funobactam, imipenem, cilastatin, and the internal standards (funobactam-15N-13C3 [Bellen, lot no. 60401-My-026], imipenem-d4 [ALSA CHIM, lot no. MS-ALS-17-241-P2], and cilastatin-15N-d3 [Cayman, lot no. 0573551]) for both matrices. Pre-treated human BAL was used as a blank matrix. Human BAL is pretreated by mixing one part of stabilizer (25:25:50 ethylene glycol/water/1 M pH 6.0 MES) with one part of human BAL (1:1, vol/vol). Pre-treated human BAL was spiked with the appropriate concentration of working standard solution to give the calibration standards and QC samples. After shaking and centrifuging, 20 μL of the supernatant was transferred into a plastic injection vial containing 300 μL of water, and then the vial was capped and vortexed for 30 s. A total of 25 μL–50 μL of injection volume was used for LC-MS/MS analysis. For the cell pellet samples, the cells were resuspended with 1 mL of Nanopure water, vortexed, and carried through three freeze-thaw cycles. After the third cycle, macrophage samples were extracted by the same procedure as for BAL fluid.

The standard curves were linear for funobactam (*R*^2^ ≥ 0.9937), imipenem (*R*^2^ ≥ 0.9929), and cilastatin (*R*^2^ ≥ 0.9960) over the concentration ranges of 2 to 1,000 ng/mL. The respective precision and accuracy for funobactam QC samples were 3.78% and 2.85% at 6 ng/mL, 2.88% and 5.99% at 300 ng/mL, and 2.93% and 4.88% at 750 ng/mL. The respective precision and accuracy for imipenem QC samples were 6.83% and 2.43% at 6 ng/mL, 4.04% and 1.31% at 300 ng/mL, and 2.91% and 2.51% at 750 ng/mL. The respective precision and accuracy for cilastatin QC samples were 11.02% and 3.90% at 6 ng/mL, 1.68% and 5.83% at 300 ng/mL, and 1.23% and 2.60% at 750 ng/mL. The LLOQ for all drugs and matrices was 2 ng/mL. Reported concentrations were multiplied by a factor of 2 to account for the 1:1 dilution of BAL fluid samples with stabilizer.

### Determination of urea concentrations

The concentrations of urea in plasma and BAL fluid supernatants were determined using LC-MS/MS at Keystone Bioanalytical (North Wales, PA). A total of 20 human plasma and 20 BAL fluid samples were assayed for urea concentrations. A total of 5 samples for both plasma and BAL fluid (25.0%) were selected for ISR testing, and the calculated assay variability values were within 20%.

The calibration range of the urea assay for plasma was linear (*R*^2^ ≥ 0.9957) over a range of concentrations from 100 to 3,000 μg/mL. The respective precision and accuracy of urea QC samples in plasma were 1.15% and 5.01% at 300 μg/mL, 1.70% and 3.41% at 1,000 μg/mL, and 1.51% and −5.15% at 2,250 μg/mL. The LLOQ for urea in human plasma was 100 μg/mL. The calibration range of the urea assay for BAL fluid was linear (*R*^2^ ≥ 0.9966) for the range of concentrations from 0.2 to 10 μg/mL. The respective precision and accuracy for urea QC samples in BAL fluid were 3.11% and −3.50% at 0.6 μg/mL, 0.76% and 1.30% at 3.0 μg/mL, 1.14% and −3.92% at 7.5 μg/mL, and 0.58% and −2.21% at 30 μg/mL. The LLOQ of urea in human BAL fluid was 0.2 μg/mL.

### Pharmacokinetic analysis

Noncompartmental methods were used to calculate pharmacokinetic parameters for funobactam, imipenem, and cilastatin in plasma. Maximum plasma concentration (*C*_max_), time to *C*_max_ (*T*_max_), and minimum plasma concentration (*C*_min_) were read from the observed plasma concentration-time profile after the start of the fifth dose of funobactam, imipenem, and cilastatin. The *C*_min_ was defined as the lowest plasma concentration during the 6 h dosing interval and after the fifth dose was administered. Area under the plasma concentration-time curve (AUC) from time 0 to 6 h (AUC_0–6h_) and from zero to the last quantifiable concentration (AUC_0–t_) after the fifth intravenous dose administration was computed using the linear-log trapezoidal method, and the AUC from zero to infinity (AUC_0–∞_) was calculated as AUC_0–t_ + *C*_last_/λ_z_, where *C*_last_ and λ_z_ are the last quantifiable concentration and terminal phase elimination rate constant, respectively. Elimination half-life (t_1/2_) was calculated as ln2/λ_z_. The apparent plasma clearance (CL) was calculated as dose/AUC_0–∞_, and volume of distribution during the terminal phase (V_z_) was calculated as CL/λ_z_. All the PK parameters were calculated by Phoenix WinNonlin, Version 8.3 (Certara USA, Inc., Princeton, New Jersey).

### Calculation of ELF volume and antibiotic concentrations in ELF and AM

The calculations of the ELF volume and drug concentrations in ELF and AM were performed with BAL fluid supernatant and cell pellet from pooled aspirates recovered from the second, third, and fourth instillations.

The concentrations of funobactam, imipenem, and cilastatin (ABX_ELF_) in the ELF were determined as follows: ABX_ELF_ = ABX_BAL_ × *V*_BAL_ / *V*_ELF_, where ABX_BAL_ is the measured concentration of funobactam, imipenem, and cilastatin in BAL fluid, *V*_BAL_ is the volume of aspirated BAL fluid, and *V*_ELF_ is the volume of ELF sampled by the BAL. *V*_ELF_ is derived from the following: *V*_ELF_ = *V*_BAL_ × Urea_BAL_ / Urea_P_, where Urea_BAL_ is the concentration of urea in BAL fluid and Urea_P_ is the concentration of urea in plasma.

The concentrations of funobactam, imipenem, and cilastatin in the alveolar macrophages (ABX_AM_) were determined as follows: ABX_AM_ = ABX_PELLET_/ _VAC_, where ABX_PELLET_ is the measured amount of funobactam, imipenem, and cilastatin in the cell suspension, and *V*_AC_ is the volume of alveolar macrophage cells in the cell suspension. The ABX_AM_ was derived by adjusting for the percentage of macrophages in AM using the differential cell count of the BAL fluid. *V*_AC_ was determined by multiplying the cell count in BAL fluid by the mean macrophage cell volume of 2.42 µL/10^6^ cells (27).

The ratios of ELF and AM concentrations to the simultaneous plasma concentrations were calculated for each subject and summarized per group at each sampling time. The mean concentration values of funobactam, imipenem, and cilastatin at each BAL sampling time (e.g., 1, 2, 4, and 6 h) were calculated and used as a single data set to estimate the AUC_0–6h_ of plasma, ELF, and AM. The concentration at the 6 h sampling time also served as a value at time zero for determining the AUC_0–6h_ of plasma, ELF, and AM. The AUC_0–6h_ for each matrix was determined with the linear-log trapezoidal method using noncompartmental methods, as noted above. The ratio of concentration of ELF and AM to the unbound plasma concentrations at each sampling time, and the ratio of AUC_0–6h_ of ELF and AM to that of unbound plasma, were calculated using an unbound plasma fraction of funobactam of 0.813. The unbound plasma fractions used for imipenem and cilastatin were 0.80 and 0.60, respectively (28).

### Laboratory and safety assessment

All subjects who received at least one dose of funobactam or imipenem/cilastatin were included in the evaluation of safety, including clinical laboratory tests (serum chemistry, hematology, and urinalysis), ECG, physical examination, and vital signs, as well as adverse event monitoring. The adverse event data were primarily presented by system organ class, the relationship to the study medication, and severity. For other safety data, the observed data and/or the changes from baseline were summarized by descriptive statistics.

## SUPPLEMENTAL MATERIAL

Supplemental tables aac.00524-26-s0001.docx Tables S1 to S4.

ASM does not own the copyrights to Supplemental Material that may be linked to, or accessed through, an article. The authors have granted ASM a non-exclusive, world-wide license to publish the Supplemental Material files. Please contact the corresponding author directly for reuse.
