# Supplementary material for: Plasma and intrapulmonary pharmacokinetics of funobactam, a novel diazabicyclooctane β-lactamase inhibitor, administered in combination with imipenem/cilastatin in healthy subjects
Source: Antimicrob Agents Chemother. 2026 Jun 15;70(8):e00524-26. doi: 10.1128/aac.00524-26 (PMC13435829; doi:10.1128/aac.00524-26)
Supplement: Supplementary file 1 [file aac.00524-26-s0001.docx]

**Supplementary Tables**

**Plasma and Intrapulmonary Pharmacokinetics of Funobactam, a Novel Diazabicyclooctane β-lactamase Inhibitor, Administered in Combination with Imipenem/Cilastatin in Healthy Subjects**

**Runing Title:** Plasma and intrapulmonary PK of funobactam and imipenem

**Authors**

Keith Rodvold^a^, Mark Gotfried^b^, Aramayis Kocharyan^c^, Grigor Mamikonyan^c^, Qifeng Shi^d^, Hui Zhao^d^, Allan Xu^e^, Meijie Le^d^, Jianguo Li^f^

^a^University of Illinois Chicago, Chicago, Illinois, USA

^b^Pulmonary Associates, PA, Phoenix, Arizona, USA

^c^Clinartis LLC, Hollywood, Florda, USA

^d^Evopoint Biosciences Co., Ltd., Suzhou, China

^e^Keystone Boanalytical, LLC, North Wales, PA, USA

^f^Evopoint Biosciences USA, Inc., MA, USA

**Table S1.**	Characteristics of 20 nonsmoking, healthy adult subjects receiving 60-minute intravenous infusions of 250 mg XNW4107 in combination with 500 mg imipenem/500 mg cilastatin every 6 hours for five doses

|  |  |  |  |  |  | Bronchial Lavage Fluid (BAL) | |
| --- | --- | --- | --- | --- | --- | --- | --- |
|  | Sex | Age  (years) | Height  (cm) | Weight  (kg) | CL_CR_  (mL/min/1.73m^2^) | Total WBC in  BAL Fluid  (cells/mm^3^) | Macrophages  (%) |
| All Subjects^a^ | 13 M, 7F | 43 ± 8 | 172 ± 8 | 80.3 ± 11.5 | 95 ± 19 | 146 ± 97 | 78 ± 8 |
| 1-hour Cohort^b^ | 3 M, 2F | 40 ± 14 | 172 ± 6 | 77.6 ± 14.5 | 99 ± 18 | 144 ± 100 | 79 ± 5 |
| 2-hour Cohort^b^ | 3 M, 2F | 44 ± 6 | 173 ± 8 | 78.5 ± 13.5 | 93 ± 14 | 112 ± 47 | 77 ± 13 |
| 4-hour Cohort^b^ | 3 M, 2F | 42 ± 5 | 173 ± 6 | 83.4 ± 9.7 | 97 ± 26 | 167 ± 155 | 78 ± 11 |
| 6-hour Cohort^b^ | 4 M, 1F | 48 ± 5 | 171 ± 12 | 81.6 ± 10.9 | 90 ± 20 | 162 ± 77 | 79 ± 4 |

Data are expressed as arithmetic mean ± SD except for sex (presented as number of subjects)

^a^ 20 subjects (pharmacokinetic population)

^b^ 5 subjects per cohort

M = male; F = female

CL_CR_ = Calculated creatinine clearance based on Cockcroft-Gault equation

**Table S2.** Funobactam concentrations in plasma, epithelial lining fluid (ELF), and alveolar macrophages (AM) at assigned BAL sampling times

| BAL Sampling  Time | Plasma (total)  (µg/mL) | ELF  (µg/mL) | AM  (µg/mL) |
| --- | --- | --- | --- |
| 1-hour^a^ | 17.216 ± 2.239 | 4.426 ± 1.970 | 2.193 ± 2.791 |
| 2-hour^a^ | 11.430 ± 2.856 | 2.865 ± 0.545 | 3.796 ± 4.707 |
| 4-hour^a^ | 3.891 ± 0.978 | 1.417 ± 0.339 | 1.436 ± 0.318 |
| 6-hour^a^ | 2.499 ± 0.864 | 0.918 ± 0.345 | 1.073 ± 0.779 |

Data are expressed as arithmetic mean ± SD

^a^ 5 subjects per sampling time

**Table S3.** Imipenem concentrations in plasma, epithelial lining fluid (ELF), and alveolar macrophages (AM) at assigned BAL sampling times

| BAL Sampling  Time | Plasma (total)  (µg/mL) | ELF  (µg/mL) | AM  (µg/mL) |
| --- | --- | --- | --- |
| 1-hour^a^ | 25.171 ± 2.203 | 5.623 ± 1.926 | 1.979 ± 3.082 |
| 2-hour^a^ | 13.232 ± 6.009 | 2.885 ± 0.404 | 2.089 ± 2.193 |
| 4-hour^a^ | 2.431 ± 0.674 | 1.004 ± 0.369 | 0.360 ± 0.120^b^ |
| 6-hour^a^ | 0.921 ± 0.39 | 0.446 ± 0.175 | 0.352 ± 0.068^c^ |

Data are expressed as arithmetic mean ± SD

^a^ 5 subjects per sampling time

^b^ 4 of 5 subjects had cell pellet concentrations above quantification limits (BQL) of 2 ng/mL

^c^ 2 of 5 subjects had cell pellet concentrations above quantification limits (BQL) of 2 ng/mL

**Table S4.** Cilastatin concentrations in plasma, epithelial lining fluid (ELF), and alveolar macrophages (AM) at assigned BAL sampling times

| BAL Sampling  Time | Plasma (total)  (µg/mL) | ELF  (µg/mL) | AM  (µg/mL) |
| --- | --- | --- | --- |
| 1-hour^a^ | 24.721 ± 4.177 | 2.725 ± 1.032 | 0.922 ± 1.252 |
| 2-hour^a^ | 11.033 ± 7.132 | 1.123 ± 0.299 | 1.371 ± 1.800 |
| 4-hour^a^ | 1.138 ± 0.433 | 0.484 ± 0.428 | 0.376 ± 0.225^b^ |
| 6-hour^a^ | 0.406 ± 0.191 | 0.252 ± 0.089^c^ | 0.252 ± 0.177^d^ |

Data are expressed as arithmetic mean ± SD

^a^ 5 subjects per sampling time

^b^ 4 of 5 subjects had cell pellet concentrations above quantification limits (BQL) of 2 ng/mL

^c^ 3 of 5 subjects had BAL concentrations above quantification limits (BQL) of 4 ng/mL

^d^ 3 of 5 subjects had cell pellet concentrations above quantification limits (BQL) of 2 ng/mL
